# Drivers of Automation and Consequences for Jobs in Engineering Services: An Agent-Based Modelling Approach

**DOI:** 10.3389/frobt.2021.637125

**Published:** 2021-05-10

**Authors:** Hildegunn Kyvik Nordås, Franziska Klügl

**Affiliations:** ^1^ Economics and Statistics, School of Business, Örebro University, Örebro, Sweden; ^2^ Council on Economic Policies (CEP), Zürich, Switzerland; ^3^ Machine Perception and Interaction Lab, AASS, School of Science and Technology, Örebro University, Örebro, Sweden

**Keywords:** technology uptake, employment, automation, economic modelling, agent-based simulation

## Abstract

New technology is of little use if it is not adopted, and surveys show that less than 10% of firms use Artificial Intelligence. This paper studies the uptake of AI-driven automation and its impact on employment, using a dynamic agent-based model (ABM). It simulates the adoption of automation software as well as job destruction and job creation in its wake. There are two types of agents: manufacturing firms and engineering services firms. The agents choose between two business models: consulting or automated software. From the engineering firms’ point of view, the model exhibits static economies of scale in the software model and dynamic (learning by doing) economies of scale in the consultancy model. From the manufacturing firms’ point of view, switching to the software model requires restructuring of production and there are network effects in switching. The ABM matches engineering and manufacturing agents and derives employment of engineers and the tasks they perform, i.e. consultancy, software development, software maintenance, or employment in manufacturing. We find that the uptake of software is gradual; slow in the first few years and then accelerates. Software is fully adopted after about 18 years in the base line run. Employment of engineers shifts from consultancy to software development and to new jobs in manufacturing. Spells of unemployment may occur if skilled jobs creation in manufacturing is slow. Finally, the model generates boom and bust cycles in the software sector.

## 1 Introduction

Due to recent advances in algorithms and technology based on Artificial Intelligence (AI), intelligent automation systems are rapidly moving into the workplace. AI technologies such as Deep Learning have become accessible to industry as a result of growing digitisation, the consequent availability of data, computation power and powerful tools, propelled by research by the leading technology companies. Nevertheless, the adoption of technology is gradual, often with long lags between innovation and adoption^1^. A survey of firms’ use of AI released by Statistics Sweden in November 2020, for example, finds that only 5.4% of the firms surveyed use AI. In the United States, the 2018 Annual Business Survey found that 10.3% of firms use at least one of the advanced business technologies classified as AI^2^. Against this backdrop, it is clear that to assess the impact of AI on the future of work, one first needs to understand what determines the uptake of AI in firms. This paper contributes to filling this gap. It studies the uptake of AI-based automation and its determinants as a market interaction between developers and users of AI-based automation software.

It contributes to the literature in three major ways.

First, studies on the adoption of AI are few despite the observed long lags between innovation and adoption. It is well documented in the literature that adoption of new technology goes together with investment in intangible assets, including skills and organisational innovations (Brynjolfsson and Hitt, 2000; Rock, 2019). Nevertheless, standard models of technology adoption do not feature organisational changes. Our novel approach contributes to filling this gap by modelling AI-adoption as a switch in business model. Before AI-adoption, engineers serve their manufacturing clients through face-to-face, on-site interaction. AI-adoption implies using AI technology to automate the engineering tasks. Among the technologies we have in mind are machine learning, intelligent planning, automated reasoning, text mining and natural language generation. Many of these AI approaches have become applicable outside purely academic contexts due to accessible tools and platforms^3^. These automation technologies are embedded in software, such as intelligent systems for computer-assisted design (CAD), systems supporting additive manufacturing (3D printing), software for advanced construction of digital twins, software performing advanced data analysis and complex tests for verifying control software. Engineers switch from a consultancy model to developing, maintaining and licensing such software to clients. Manufacturers switch to a more skills-intensive software-supported production technology. The driving forces that we analyse are economic and institutional, notably uncertainties about user costs and benefits of the new technology, the switching costs to a different business model, the need for skills upgrading, and regulatory incentives or disincentives. Our focus on the demand side of technology diffusion provides new insights that can inform a balanced R&D, skills- and labour market policy.

Second, the study focuses on AI-adoption in services, noting that professional services are at the cusp of using AI-enabled automation^4^. Indeed, the Swedish AI adoption survey found that services sectors that produce and use ICT intensively have the highest AI-adoption rate in the economy. While AI is on its way into most professional services, engineering has a long history of developing technology for modern manufacturing, for instance through computer assisted design (CAD) feeding into computer assisted manufacturing (CAM). Here, with advanced image processing and new approaches combining data-driven learning and (spatial) reasoning, AI-based software can automate knowledge-intensive services previously performed by specifically educated engineers. The vision of Industry 4.0 (Lasi et al., 2014; Wang et al., 2018; Rock, 2019) further drives these developments. Today, civil engineers top the list of occupations most affected by AI while three other engineering occupations feature among the top 20 (Felten et al., 2019). Despite the susceptibility to automation, engineers are among the occupations with the fastest job growth in recent years^5^. Engineering is therefore of particular interest for understanding the relationship between AI and jobs in high-skilled services occupations.

A recent EU enterprise survey on the use of technologies based on AI found that about 60% of AI-using firms buy software or ready-to-use systems from external services suppliers^6^. Our modelling strategy reflects this empirical observation. Thus, engineering firms are the external suppliers of AI-enabled software and ready-to-use systems, engaging in market interactions with manufacturers. Most existing studies focus on the impact of robotics for automation in manufacturing. One reason for this is that while data on robot use is readily available, data on AI-enabled software use is not.

This leads to our third major contribution, which is to develop an agent based model (ABM) to study the joint adoption of AI in services and manufacturing. ABMs are particularly suitable for dynamic processes where outcomes are uncertain and agents interact (Dawid, 2006). Furthermore, it is apposite when the future is likely to be qualitatively different from the past such as during technological transitions (Farmer and Foley, 2009). Our ABM captures the interactions between the agents and the environment in which they operate and generates important insights on the trajectory of AI adoption. Notably, the model generates the boom and bust cycles often observed during the early stages of technology adoption. The combination of traditional economic modelling and the rigorous agent-based perspective used in our study results in a rather complex, yet comprehensive model. Using a stringent agent-based perspective, we avoid the “invisible hand” that automatically and instantly clears markets. Instead agents decide strictly based own experience, perception and individual economic reasoning, allowing us to trace out the process of technology adoption step by step. Agent-based approaches to economic modelling per se are not new (Tesfatsion, 2006; Hamill and Gilbert, 2016; Gatti et al., 2018), yet still far from being a mainstream approach in economic modelling.

Our model has two types of agents, engineering firms and manufacturing firms; and two business models, which we label consultancy and software respectively. Consultancy is the traditional business model where engineering firms deploy consultants to clients, working with them on-site and face-to-face to solve problems. In the software model consultants are replaced by in-house engineers working with intelligent systems for automating engineering services. Manufacturers buy such software through licensing agreements, paying a license fee, or they may opt for cloud-based software-as-a-service, paying an annual subscription rate. The model generates a change of business model when engineers have gathered sufficient experience to create software solutions that automate services that were previously provided by consultants. Gathering this experience is modelled as learning-by-doing and represents dynamic economies of scale. Manufacturers decide whether to license software or stick to the consultancy model based on the expected costs and benefits of doing so. The benefits are uncertain at the time of the decision. Our analysis shows that it is hesitance on the part of manufacturers that holds back the uptake of AI-based software.

The rest of the paper is organised as follows: Section two discusses related research while section three develops a conceptual framework that captures the interaction between engineering firms, their clients and the environment in which they operate. The framework is coded into a dynamic ABM in section four. Section five presents the simulation results, while section six summarises and concludes.

## 2 Relations to Previous Work

The literature on adoption of AI in the workplace is new and to the best of our knowledge this is the first paper to simulate the adoption of AI in business services. It builds on the theoretical literature on technology diffusion and adoption pioneered by Nelson and Phelps (1966); Rosenberg (1972); Davies and Davies, (1979); Stoneman and David (1986) and others. The theory is inspired by the stylised fact that the adoption of new technology follows an S-curve with slow uptake at an early stage, followed by a sharp rise in adoption when a critical mass is reached, until the market is saturated and the curve flattens (Gort and Klepper, 1982; Hall and Khan, 2003)^7^.

Two different classes of theoretical models can explain such a pattern. The first envisages technology diffusion as the propagation of information, using models similar to those explaining epidemics. Observing that technology spreads much slower than epidemics and information, a learning process is added to the theory. Thus, firms learn by using new technology, and some of the accumulated tacit knowledge enters the public domain over time (Rogers, 1995).

The other major theory of technology adoption focuses on the characteristics of early technology adopting firms. Such models feature differences in firm size, productivity and abilities as explanatory variables. A new technology is fraught with uncertainty about its potential benefits, which introduces expectations as an important component of the theory. Furthermore, to reap the full benefit from a new technology, complementary investments in skills, reorganisation of production and rearranging relations to suppliers and customers are needed^8^. Therefore, the largest, most productive or otherwise most capable firms adopt new technology first (Davies and Davies, 1979; Rogers, 1995).

Recent survey data from the US and Sweden finds that it is indeed the largest and most productive firms that adopt AI. This explains the early, slow diffusion part of the S-curve. The subsequent acceleration in uptake may stem from standardisation of the technology as experience with using it accumulates, substantially reducing uncertainty over time. Network effects can also be important when the benefits from adopting the technology depends on suppliers or customer adopting it too. Then, the switching cost to the new technology declines as the number of users increases. Our model builds on the second strand featuring firms that differ in productivity, uncertainty about the benefits of new technology and switching costs. Our model also features network effects as well as learning by doing that reduces uncertainty and adoption costs over time. It generates the S-curve predicted by the theoretical literature in a setting of interaction between supply and demand and technology that has the features of AI-driven software, i.e. substituting for skilled workers, high cost of software development but zero marginal cost of adding another user (Varian, 2019).

Turning to the literature on technology and jobs, the most common approach to studying the impact of AI-related technology on jobs is to break jobs down to tasks and analyse the task content of different occupations (Autor et al., 2003; Acemoglu and Restrepo, 2018; Neves et al., 2019). The approach is to identify tasks that can be automated, vs. tasks that complement AI, and make predictions about the future of work from these metrics. In our context, this would generate business models where engineers may offer both software and consultancy, or it could generate deeper specialisation in the engineering sector where automatable tasks are performed by software while new tasks are performed by engineering consultants. However, this literature assumes that all tasks that can be automated are automated instantly, and thus assumes away adoption costs. Our contribution to the literature is precisely to focus on the scenario where existing technology is not instantly adopted, which is clearly the empirically most relevant case. The scenario is mentioned in Acemoglu and Restrepo (2018), but is not further developed. We explore and endogenise the uptake of technology as a function of wages, the cost of switching to AI-driven software, including the cost of reorganising production, and the expected gains from switching to new technology. Our model also features reallocation of engineering jobs across activities from consultancy to software development and maintenance, and to employment in manufacturing where engineers work on technical problem solving using AI-driven software.

On methodology, our paper relates to Agent-based Modelling and Simulation that has become an established micro-simulation approach in social sciences, economics (Gallegati and Richiardi, 2009; Hamill and Gilbert, 2016), ecology and for modelling complex systems in general (Klügl and Bazzan, 2012). The underlying metaphor of such a model is a set of interacting agents—that can be basically seen as situated intelligent, autonomous actors (Wooldridge, 2009). A model captures agents’ decision making in their individual environmental context which may be changing and influenced by multi-level feedback loops. During simulation, overall dynamics are generated. Consequently, agent-based simulation is particularly apt for modelling endeavours which involve heterogeneous agents, with transient dynamics and without the necessity of an equilibrium-based model. Technology adoption, which has all these features, is best understood through the lens of interacting agents. Our paper integrates insights from economics and Agent-Based Modelling by assigning decision making rules from economic theory to individual interacting agents within the framework of an ABM.

## 3 The Model

### 3.1 Intuition

We propose a dynamic model consisting of two types of agents: engineering firms and their manufacturing clients. Manufacturers produce final goods according to a production function which combines production workers employed by the manufacturer and services inputs sourced from engineering firms. We distinguish two types of relationships between the engineering firms and the manufacturer: consultancy and software.

The consultancy model involves engineers working with the client, on-site, face to face, to solve problems and provide necessary services for production. The problems and services are client-specific and the ability to solve them rests with the consultant. The engineering firm and the manufacturer enter a contract which specifies the tasks the consultants are to perform as well as the payment, which is an annual fee per consultant. Contracts are setup anew every year; the number of consultants needed depends on the productivity and size of the manufacturer. Engineers are also explicitly modelled as discrete entities with individual experience that increases when working for a highly productive manufacturer.

In the software model the engineering firm establishes an R&D department where assigned engineers develop software that automates services adopting available AI technology such as machine learning or reasoning based on the problem solving experience of the engineers. The R&D activity requires a given number of engineers; their salaries constitute a fixed cost which the engineering firm recuperates through the licensing of the resulting software. Once developed, the software can be licensed to an unlimited number of manufacturers.

Each engineering firm offers its unique variety of the service, and thus distinguishes its product from competitors. Such product differentiation implies that the engineering firms may charge customers a premium and mark up their price over marginal cost. In the case of occupational licensing, engineers have exclusive rights to perform a predefined set of tasks. Furthermore, they may limit the number of licensed engineers and thereby charge a higher mark-up.

Manufacturers are heterogeneous in terms of size and productivity. Productivity is a measure of how effectively the firm transform inputs into outputs. Thus, the more productive firms use less engineering services per unit of output. Switching business model from relying on external consultants to using software, supported by in-house engineers, involves restructuring of production for a seamless interface between fabrication and the software. This requires upgrading of machinery and skills, creating jobs for engineers to manage the interface between the software and machinery, supervise production workers, support management in technical decision making, and govern the licensing contract with the engineering firm^9^. The dynamics of the model consist of learning by doing on the part of engineers working on problem-solving in manufacturing firms and network effects in the adoption of software.

### 3.2 Formal Model

Manufacturers, indexed *i*, are heterogeneous in terms of productivity denoted $\theta_{i}$, which follows a Pareto distribution. The probability density function of the Pareto distribution is given by $g\left(\theta \right)=k{\left(\theta_{\min}\right)}^{k}{\left(\theta \right)}^{-\left(k+1\right)}$ where $\theta_{\min}$ is the scale parameter, which we set to unity, and *k* is the shape parameter, which we tentatively set to 2.2^10^. The corresponding cumulative density function is $1-{\left(\theta_{\min}/\theta \right)}^{k}$. The manufacturers produce final output, denoted *Y* using production workers indexed *l* and engineering services. Total costs for the consultancy and software models respectively at time *t* are:

$$TC_{i,c}=\left[\frac{w_{i}}{\alpha }+\frac{\varphi w_{s}}{\beta_{c}\theta_{i}}\right]Y_{i}$$ (1)

$$E_{t}\left[TC_{i,softw,t}\right]=\frac{A_{t}}{\theta_{i}}w_{l}^{1-\beta }w_{s}^{\beta }Y_{i}+\delta +\gamma$$ (2)

*TC* represents total cost of production. The two business models are indexed *c* and *softw* respectively. In both cases we apply constant elasticity of substitution production- and cost functions. In the consultancy model we use the extreme case of a Leontief specification where production factors are perfect complements, while in the software model we apply the Cobb-Douglas functional form where the elasticity of substitution between factors is unity. These particular functional forms are not critical for the results, but serve to distinguish between more and less flexible technologies in the two business models.

**FIGURE 1:**
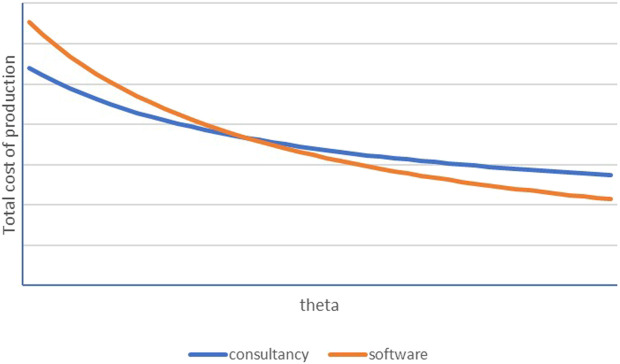
Total cost comparison, consultancy and software.

Variables and parameters: *α* represents the production worker intensity while $\beta_{c}$ depicts the consultancy input intensity of manufacturing production in the consultancy business model. Wage rates for production workers and engineers are denoted by $w_{l}$ and $w_{s}$ respectively, while the mark-up rate that engineering firms obtain for their consultancy services is *φ*. A scale parameter *A*, the license fee for software *δ*, and a stochastic element *γ* are additional parameters in the cost function for manufacturers that opt for the software model. The stochastic element *γ* is normally distributed $\gamma ∼N\left(0,\sigma^{2}\right)$. Manufacturers will be in the market for software if the expected cost of switching to software is lower than continuing with the consultancy model. Figure 1 illustrates the two cost functions where the horizontal axis represents manufacturing firms’ productivity and the vertical axis total cost. Clearly, software represents the lowest cost for high-productivity firms, while consultancy is the better option for low-productivity manufacturers^11^.

There are network effects related to the switch to the software model as adopters reorganise production, including relations to suppliers and customers around the software. Also professional organisation’s investment into competence development speeds up technology adoption. We capture this by modelling the scale parameter *A* to be a declining function of the number of firms that have switched to software. The network effect works with one period lag.

$$A_{t}=\frac{A_{t-1}}{n_{softw,t-1}^{\mu }}$$ (3)

where 0<μ<1. Demand for engineering consultancy services from each manufacturing firm choosing that model is given by:

$$C_{i}=\frac{Y_{i}}{\beta_{c}\theta_{i}}$$ (4)

Manufacturers that have switched to the software model will seek to employ engineers according to the demand function:

$$S_{i,t}=\frac{A_{t}}{\theta_{i}}{\left[\frac{\beta }{1-\beta }\frac{w_{l}}{w_{s}}\right]}^{\beta }Y_{i}$$ (5)

Engineering firms, indexed over *j* hire engineers which are deployed to client firms on a contractual basis in the consultancy model. The contract covers one period and its value varies across clients, depending on their size and productivity as indicated in the demand function, Eq. 4. The engineering firms incur wage costs only and they sell consultancy services with a mark-up factor of *φ* > 1. The consultancy revenue is thus $\varphi w_{s}\sum C_{i}$. We choose units such that one unit of consultancy services corresponds to the input of one full-time consultant for one period. Profits from the consultancy model at time *t* are:

$$\pi_{c,j,t}=\left(\varphi -1\right)\sum C_{i,t}$$ (6)

where the number of manufacturing clients changes over time. In the software model, engineering firms establish an R&D department and divert $S_{F}/\lambda_{j,t}$ engineers to staff it. The R&D department uses available data sets and experience from previous consultancy efforts to create AI-based software that provides the services that are otherwise done by consultants. Thus, engineers accumulate experience from working with clients, and harness this experience into software that automates the consultants’ work.

It is assumed that a minimum number of experienced engineers is needed to successfully develop the software. So, experience accumulated over years of working with clients is an advantage when developing software, assuming that experience helps to identify appropriate machine learning architectures and to formalise knowledge for automated reasoning. We model this by introducing the experience of the engineer, denoted *λ* in the cost of developing the software. The total cost of switching for the engineering firm is the wage costs for the engineers working in the R&D department and the foregone profits from no longer deploying them to clients as consultants. Revenue in the software model will be the licence fee *δ* times the number of manufacturers that license the software from company *j*; $n_{softw,j,t}\delta$. Expected profits from the software model at time *t* is thus:

$$E_{t}\left[\pi_{softw,j,t}\right]=E_{t}\left[n_{softw,j,t}\right]\delta -\frac{w_{s}S_{F}}{\lambda_{j,t}}\varphi$$ (7)

The engineering firm knows the cost of developing software, but at the point of decision whether to develop it, the number of clients that will take up the software is unknown. The engineering firm does, however, observe the productivity of the manufacturers and thus can estimate how many of them are sufficiently productive to gain from switching to software. Engineering firms base their decision to develop software on expectations about how many clients they may capture from the mass of manufacturers that are sufficiently productive to benefit from switching to the software model.

After software is available, manufacturers that decided to switch their business model to software, randomly select engineering companies that offer software. Random selection is weighted by experience of the software provider assuming that more experienced firms produce higher quality software. Since the marginal cost of servicing another client is zero, it is conceivable that one engineering firm could corner the market.

It is clear from Eq. 7 that profits from switching to the software model are lower the higher the mark-up factor *φ*, predicting that engineering firms operating in a less competitive market, for instance a small market with occupational licensing, are less innovative than firms operating in a competitive market which limits the ability to charge a high price^12^. Engineering firms will develop the software if expected profits as defined in seven is positive.

After the initial investment into software development, the software life-cycle contains a number of periods with software maintenance. It is assumed that data-driven software is depreciating fast, and lasts for *T* periods. Each period between its development and obsolescence a fraction *ζ* of the number of engineers that are needed to develop the software, is sufficient to maintain it. After *T* periods, the engineering firm needs to invest again into full software development. We assume no influence of the age or status of the software on its licensing fee^13^.

Experience accumulates from working on-site and face to face with manufacturing clients. Furthermore, engineers gain more experience from working with the most productive manufacturers. An engineering firm *j*’s accumulated experience is thus a function of the productivity of the manufacturers it has worked with as follows:

$$\lambda_{j,t}\int_{0}^{t}f\left(\theta_{ij}\right)\;\text{d}\theta$$ (8)

These eight equations, representing supply and demand for engineering services in two business models constitute the conceptual core of the ABM. The forces that drive the adoption of software are engineers’ accumulated experience from working with clients and network effects from its adoption. What holds back the development of software is comfortable profits from the consultancy model, uncertainty about how many manufacturers will buy the software once the cost of developing it is sunk on the part of the engineering firms, and uncertainty about the gains from the switch to software on the part of manufacturers. These countervailing forces ensure a gradual adoption of software in the economy. The speed depends on the size of the economy, the endowment of production workers and engineers, the level and dispersion of productivity among manufacturing companies as well as policy-induced factors including occupational licensing and protection of intellectual property rights.

## 4 The Agent Based Model Setup

The agents and their role and actions are presented in Table 1.

**TABLE 1:** Summary of the modelled entities, their roles and activities.

Agents	Status	Role and actions	
		Consultancy	Software
Manufacturing firms	Active	Employ production workers	Employ production workers
		Enter consultancy contract	License software
			Employ engineers
		Produce final output	Produce final output
Engineering firms	Active	Employ engineers	Employ engineers
		Enter consultancy contract	Develop and maintain software
			License out software
Production workers	Passive	Work in manuf. firms	Work in manuf. firms
Engineers	Passive	Work in eng. firms	Work in any firm
Authorities	Passive	Occupational licensing, IPR protection	

**FIGURE 2:**
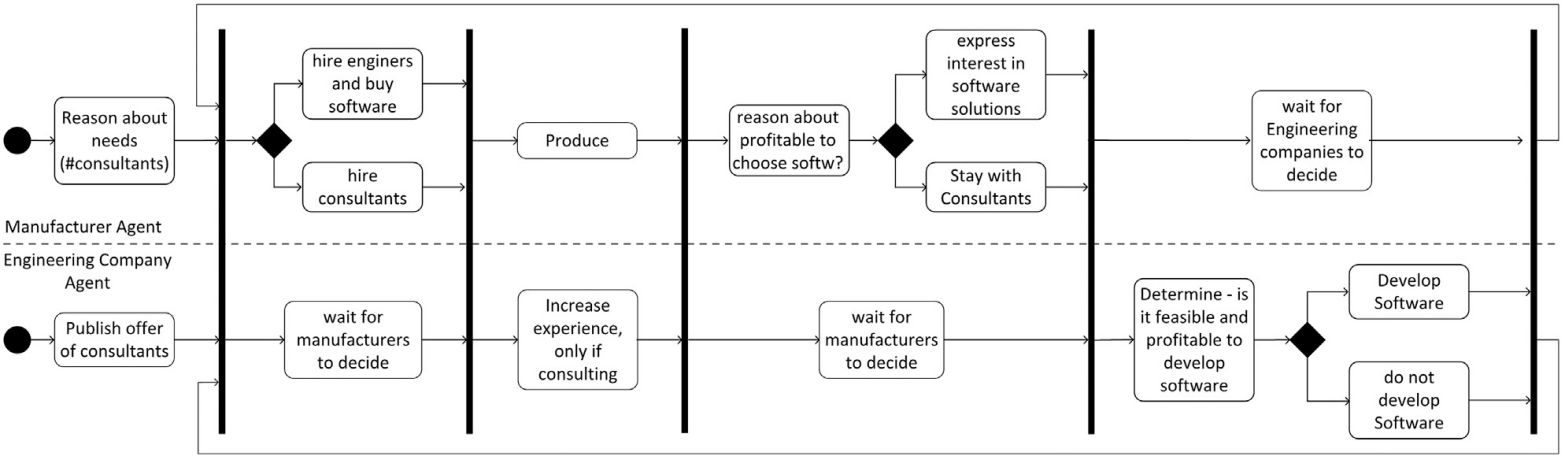
Activities of the different agent types and their coordination. Note: The black vertical lines form synchronization bars meaning that all agents need to have finished the activities before an individual agent can continue with the next activity after the bar.

The environment consists of supply of production workers and engineers, a set of exogenous parameters and decision rules as spelled out in the model presented in Section 3. All agents act in parallel and go through their individual processes within one period. Figure 2 illustrates what happens in one period including the synchronisation points between the activities that each engineering company and each manufacturer agent perform in parallel. So, manufacturer agents first determine their service needs—this happens in parallel when each engineering company either publishes their consultancy offer or offers software to be licensed (only after period two in the simulation). Then, manufacturers evaluate the offers and enter contracts or license software. After the next synchronisation step, production happens, partially with the help of consultants. The next steps with different synchronisation points are devoted to decision making for both manufacturers and engineering companies. First the manufactures reason about profitability of using software instead of hiring consultants and signal their interest. This is observed by the engineering firms who—with the information on potential size of the market for software, decide about whether they want to produce software or continue offering consultancy services. All decisions have consequences on employment of engineers.

The simulation runs through the following phases^14^:

• In phase 0—during initialisation –, manufacturer agents draw their productivity level from a Pareto distribution. Manufacturers hire production workers, which are matched to firms randomly, but the number of employees is proportional to the firms’ productivity. Engineering firms hire engineers, which are randomly matched to engineering firms.
• In phase 1 – first year—all engineering firms adopt the consultancy business model. Engineering firms and manufacturers are matched randomly and manufacturers produce final output.
• At the end of phase 1, all active firms observe their profits. Engineering firms’ experience parameter is updated. Engineering firms then consider, whether to develop software and automate their service or continue with offering consultancy services. The decision is based on expected number of clients ready to switch to the software model, and the cost of developing the software. The cost is lower for the more experienced firms. For deciding about the potential market for their software, the engineering firms observe how many manufacturer agents would be interested in software. They expect to sell to a random subset of those manufacturers who are ready to switch. If expected profits from selling software is positive, engineering firms will establish an R&D department which will work on software development. Redundant engineers, that means those not engaged in the software development, are laid off. Manufacturers decide whether actually to switch to the software model. The decision is conditioned on software being available as well as there being engineers available on the market to hire in the new jobs created during the switch to the software model. As a consequence, the most productive manufacturers are the first to switch to software. If expected profit from the software model is smaller than that for continuing with the consultancy model for all engineering firms, phase 1 is repeated and consulting engineers gain more experience during each repetition.
• In phase 2 at least one engineering firm has developed software and earns a positive profit from licensing it. In this phase the two business models coexist. Some manufacturer agents having switched business model, license software from a random supplier and hire engineers to integrate the software into the production process, other manufacturer agents continue hiring consultants. Manufacturers that do not license software and do not find consultants, do not produce output, all others do. Not all engineering firms developing software may be profitable. Making a loss from software development, causes engineering firms to immediately return to offering consultancy services.
• Manufacturers’ cost of switching to software is adjusted by the network effect given by Eq. 3. The more manufacturers use software, the cheaper it becomes for latecomers to switch, and eventually also the less productive manufacturers can afford software. Manufacturer agents who cannot recruit consultants nor can afford software, do not produce in the current cycle, but wait for opportunities in the next period. Software is maintained (bug fixes, new, minor features in small updates) at a cost $\zeta S_{F}$ with 0<ζ<1. When a software has reached obsolescence, the engineering firm decides again whether in a changed market, it could generate profit when re-developing software. Experience of engineers working as consultants is updated. Phase 2 continues until all manufacturing firms have switched to software.
• In phase 3 all firms have switched to software. There is a churning of engineering firms as software becomes obsolete and new software is developed to replace it. At this stage, engineers no longer gain experience from working directly with clients, but more are employed to support the software usage at the manufactures. There is still some dynamic ongoing at the engineering firms, as manufacturers re-select software in each period—we do not assume commitment to a particular software product. As a consequence, even when producing software in a market in which every manufacturer uses software, some software firms may lose customers to competitors, and possibly make losses on their investments.

Exogenous variables and parameters are summarised in Table 2.^15^

**TABLE 2:** Exogenous variables and parameters.

Symbol	Description	Value in the baseline case
	Number of engineering firms	30
	Number of manufacturers	100
L	Number of production workers	3000
S	Number of engineers	1000
$w_{l}$	Salary, production worker	1
$w_{s}$	Salary, engineer	1.5
α	Production worker intensity, manufacturing, consultancy model	1
$\beta_{c}$	Consultant intensity, manufacturing, consultancy model	1.5
*β*	Engineer intensity, manufacturing, software model	0.2
$\theta_{i}$	Productivity level, manufacturing firm *i*	Pareto distributed
$A_{0}$	Scale parameter, manufacturing, software model	3
*μ*	Strength of network effects of using software	0.02
*δ*	License fee, software	10
γ	Stochastic switching cost, manufacturing	normally distributed
$\lambda_{0}$	Initial experience, engineers	1
*η*	Update factor for λ	0.1
*φ*	Mark-up rate, consultancy	1.3
$S_{F}$	Number of engineers needed to develop software	18
ζ	Software maintenance cost relative to development cost	0.5

The ABM was implemented using the SeSAm platform^16^ which is a fast prototyping environment for agent-based simulations providing an activity diagram-like way of implementing complex agent behaviour.

## 5 Results

We start by running the simulations with baseline parameters as reported in Table 2, including sensitivity analysis on the overall size of the sector and the ratio of production workers to engineers. We experiment next with policy relevant parameters: 1) the mark-up rate, which is related to the strength of competition in the engineering services market and 2) the license fee, which is partly related to the strength of intellectual property rights protection and partly to the strength of competition in the market for software. Eventually, we want to explain what are the relevant factors influencing how fast intelligent automated solutions distribute in a market characterised by the parameters above as well as what is the actual impact and dynamics on the employment of highly qualified engineers.

### 5.1 Baseline

**FIGURE 3:**
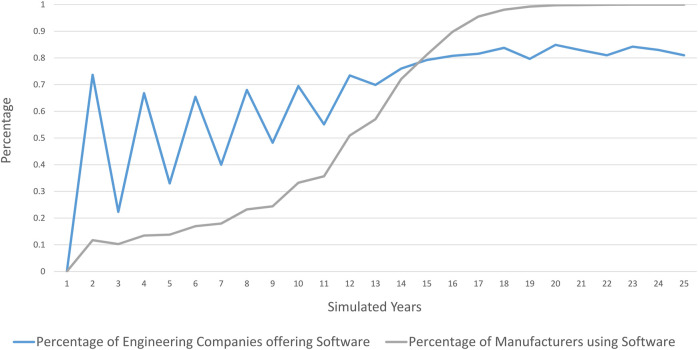
Percentage of companies having switched to the software model Note: Baseline setting as described in Table 2.

We start by simulating the baseline scenario^17^. As described, we start with a scenario where all firms are in the consultancy model. Firms next decide whether to switch to the software model and look for a supplier or customer for software. As Figure 3 indicates, a few manufacturing firms already switch to software in the second year. All engineering firms anticipate the market opportunity these firms constitute, and a large share of them decides to develop software. However, the customers are few, competition is fierce, and most early software developers fail. As a consequence, those failing firms give up to offer software^18^.

The uptake of software in manufacturing is gradual and about half of all manufacturers have switched to software after 11 years. The uptake does, however, accelerate after about a third of all manufacturers have switched, and levels off when about 90% of firms have switched^19^. During the first decade of relatively slow uptake, there is a competitive fringe of engineering firms that develops software, fails and exits as indicated by the zigzagging of the blue line in the chart. After all manufacturers have switched to the software business model, about 80% of engineering firms offer software. There remains a competitive fringe of engineering firms that exit when a loss from software happens, when a new development is necessary, but too expensive or when simply not a sufficient number of software licenses were acquired. A start-up seeks consultancy contracts, but realises that demand for such services is close to zero and quickly starts to develop software as well.

What we see in our simulation shown in Figure 3 is a largely demand-driven adoption of automation software, and a boom and bust cycle in the automation software sector. The booms are driven by all engineering firms simultaneously forming expectations about the number or clients that will shift to software (Eq. 7). However, not all software firms will find customers for their software. Those who do not, exit the software market and reestablish as consultant. This cycle is similar to the so-called *dot.com* bubble that could be observed in the 1990s when adoption of ICT took off, although in that case the financial market amplified the cycle^20^.

**FIGURE 4:**
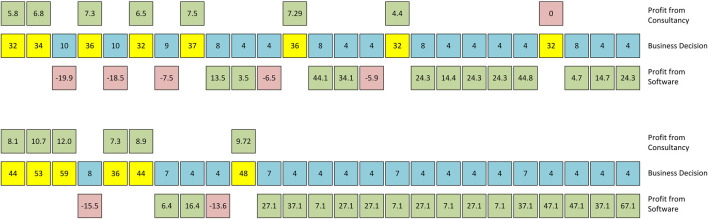
Two examples of the lifeline of randomly selected engineering firms. Note: The middle row shows the selected business model (consultancy in yellow, software in blue). Numbers in these cells denote the numbers of employed engineers in that year. The upper cells contain the profit the company made in cases of consultancy, the lower cells profit or loss when trying to sell software.

For explaining this overall behaviour, a look into the dynamics on the agent level is helpful. Figure 4 depicts the lifeline of two randomly selected engineering firm agents. They both start out as consultants and earn a positive profit. They both end up profitably licensing software, and they both have at least one unsuccessful attempt at switching to software. The first company has two spells of consultancy after a commercially successful software becomes obsolete, while the second company experiences only one such event.

**FIGURE 5:**
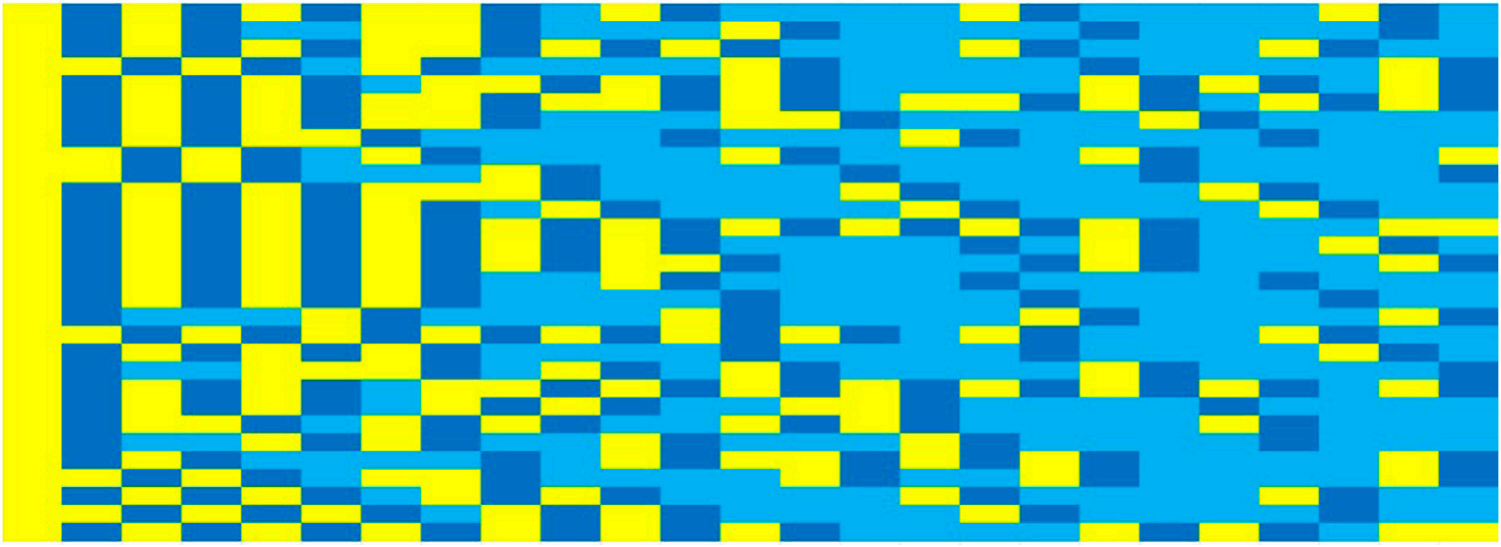
Business model dynamics of all engineering companies over 25 simulated years. Note: Yellow means that the agent offers consulting, dark blue: it develops software, blue: it maintains software.

Figure 5 shows the business model dynamics for all engineering firms over the complete simulation run. We observe that they have all entered the software model after two years, but only three are successful and continue in the third year to maintain and develop their software. As time passes, the dynamics turn increasingly toward a shifting between developing new and maintaining existing software, but all firms experience occasional failures in the market for automated software.

**FIGURE 6:**
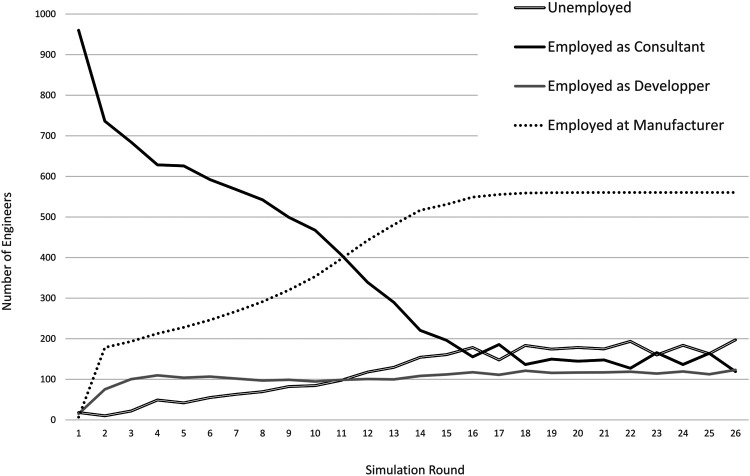
Development of employment over simulation time. Overall number of engineers is 1,000.

In addition to the technology uptake, we also want to analyse dynamics of the composition of engineer employment. Figure 6 depicts the dynamic impact of technology adoption on employment of engineers. All engineers work as consultants in the first year. Consistent with the changing business model, they gradually move to the R&D department in the engineering firm where they develop and subsequently maintain software. Consultants that cannot find a job in the R&D department are laid off. Most of them find new jobs in manufacturing firms that have switched to software and are looking for engineers to fill new jobs created during the transition to a more sophisticated and skills-intensive production process. Finally, some of the laid off engineers do not find a new job immediately, and become unemployed. We notice that with substantial economies of scale in software development, the number of workers needed to develop and maintain software is relatively small. Our simulations thus predict that most of the changes in employment are from external consultants to engineers working in manufacturing^21^.

An interesting parameter is β, the engineer intensity influencing how many engineers are needed to support complex software usage at the manufacturer (Eq. 5).

**FIGURE 7:**
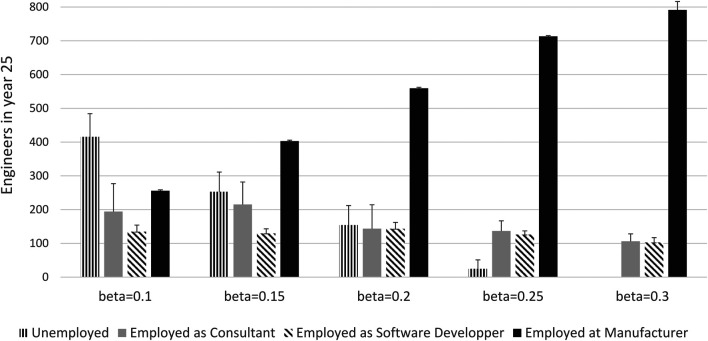
Influence of *β* on the employment structure.

The unemployment rate among engineers following the transition to software depends crucially on the ratio of production workers to engineers in the labour market and the desired skills composition of employees in manufacturing firms that have switched business model. Sensitivity analyses depicted in Figure 7 shows that there will be full employment of engineers at the end of the transition period if *β* is larger than about a quarter. Sensitivity analyses also show that with fewer engineers in the market relative to production workers there could also be shortage of engineers at lower levels of *β*. Our results reflect the S-curve of technology adoption predicted by the theoretical literature e.g. (Gort and Klepper, 1982; Hall and Khan, 2003). It is also compatible with recent shifts in employment patterns where the share of professional jobs in manufacturing has increased from 5.7 to 9.4% from 2008 to 2019 in the European Union, and the share of technicians and associate professionals have increased from 13.4 to 15.6% during the same period^22^. Finally, our results reflect work by Andrews et al. (2015) which shows that the most productive firms are the first to adopt new technology.

### 5.2 Experiments, the Mark-Up Rate

The mark-up rate reflects the strength of competition in the market for consultant engineering services. High mark-up rates may stem from occupational licensing that gives licensed engineers exclusive rights to perform a defined set of engineering tasks, a small market closed to foreign competition, or simply a shortage of engineers for instance due to low education capacity for engineers or a limited number of engineering licenses issued.

**FIGURE 8:**
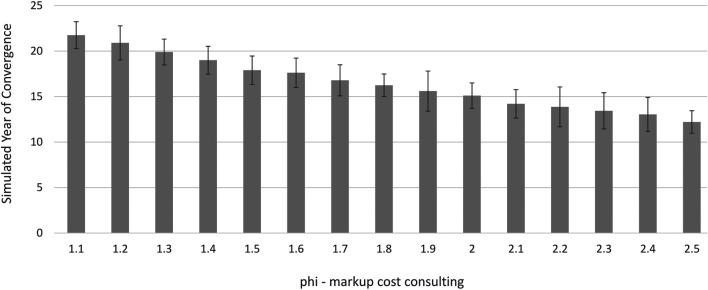
Mark-up rates and simulated year in which 100% manufacturers were using software. Note: Averaged over 30 simulation runs, standard deviation shown as error bars. In the case of φ=1.1,1.4 and 2.4 two runs was omitted as not converged. With φ=1.3,1.6,1.8 and 2.5 convergence was not reached in one single run. We consider those cases as outliers with randomly generated manufacturers that are particularly small and omitted those from the analysis.

From Eqs. 1 and 2 we see that a high mark-up rate makes consultants relatively more expensive than software. On the other hand a higher mark-up rate yields higher profits for the engineers in the consultancy model (Eq. 6). Thus, manufacturers are more likely to switch to software the higher the mark-up rate, while engineering firms are less likely to switch the higher the mark-up rate. It follows that if adoption of the software model is driven from the demand side, the adoption rate increases as the mark-up rises. If on the other hand the uptake is driven by a supply push, then we would expect it to be delayed for longer the higher the mark-up rate. Figure 8 clearly shows that this is a demand pull story.^23^

**FIGURE 9:**
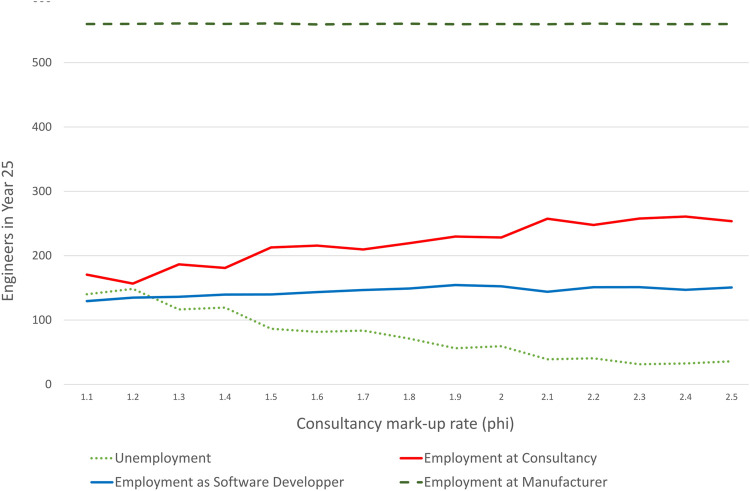
Employment in simulated year 25 depending on the consultancy mark-up rate. Averaged over 30 simulation runs.

Figure 9 shows employment of engineers by sector and activity after 25 periods as a function of the mark-up rate. We first notice that employment of engineers in manufacturing is largely unaffected by the mark-up rate. After 25 periods all manufacturers have switched to the software model and pay engineers the going wage $w_{s}$, rather than the marked-up consultancy fee, so this is no surprise. Employment as software developer is also largely unaffected by the mark-up rate. Where we do see a significant difference is on the employment of consultants and the unemployment rate for engineers. The employment as consultant is actually very brittle as there is practically no market for consultancy services. Engineering firms that made a loss with software provision, try to re-establish with consultancy, yet there is hardly any demand for consultancy services and thus the profit from consultancy is zero. Such a company lays off all the newly recruited engineers, but tries to re-recruit them again in the next cycle, when deciding about producing software or offering consultancy again. Yet, depending on the competition in recruiting engineers, the full number may not be available any more.

An important policy implication of the simulations is that the potentially harmful delay of the uptake of technology due to occupational licensing does not materialise in a demand-driven market. This conclusion holds when the mark-up rate is unrelated to the software license fee and thus, exclusive rights do not extend to software licensing. We now turn to an experiment where we let the license fee vary.

### 5.3 Experiments, the License Fee

**FIGURE 10:**
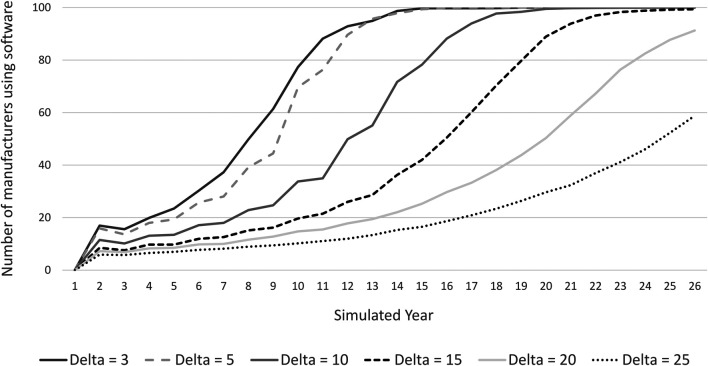
Number of manufacturers who use software over simulated time with different settings of the licence fee Delta.

As Figure 10 indicates, the adoption rate of software is slower, the higher the license fee *δ* is set. From Eq. 2 we observe that cost of production is higher for manufacturers the higher is δ, so there will be fewer takers of software the higher is *δ*. This also results in weaker network effects, further slowing down the uptake over time (see Eq. 3). On the other hand, as depicted in Eq. 7 the revenue of the engineering firms providing software is higher the higher is δ, all else equal. Thus, the slower rate of transition to the software model stems from the demand side.

A convergence toward a situation in which all manufacturers use software happens also in scenarios with high licence fees. Longer simulation runs with δ≥20 confirm that the adoption rate eventually approaches 100%. For example, with δ=25, the population of firms converges into a stable situation with slightly less than 100% of manufacturers using software between years 50 and 60. In these simulations, two engineering firms are too small to start software development and try to hold on to the consultancy model.

**FIGURE 11:**
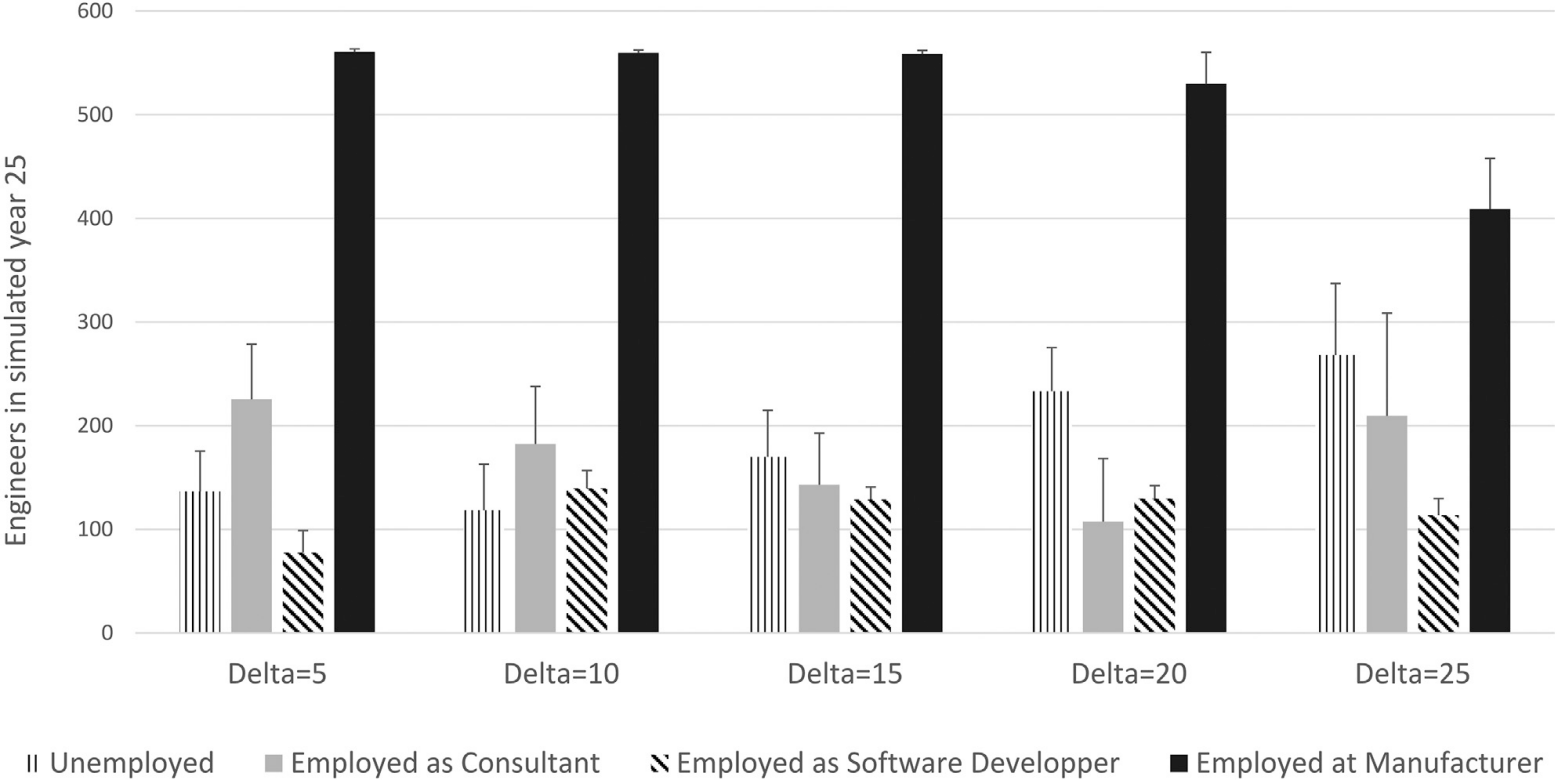
Employment of engineers in the simulated year 25. Note: Average over 30 runs, the error bar shows the standard deviation between runs. Delta is the licence. All other parameter according to baseline scenario.

A higher license fee also results in a higher rate of unemployment among engineers during and after the transition to software as illustrated in Figure 11. Consultancy jobs are lost, and job creation in the R&D department to develop and maintain software together with engineering jobs created in manufacturing is insufficient to absorb the idle consultants. However, sensitivity analyses with a higher *β* show that unemployment among engineers is substantially reduced or even eliminated when also *δ* is higher than in the baseline scenario.

**FIGURE 12:**
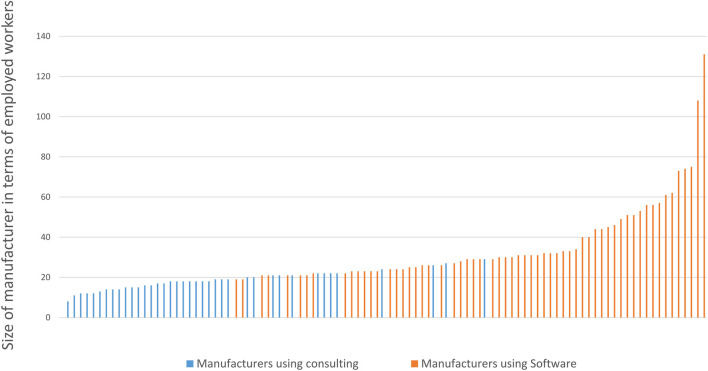
Illustration of the dependency between size of a manufacturer and its decision to use software. Note: Delta = 50, in year 25. With γ, there is a random element in the decision making: for sizes between 19 and 29 employed workers, both decisions are observed, yet with corresponding tendencies.

Finally, Figure 12 shows the number of manufacturing firms that take up software in a scenario where software is very expensive and the two business models co-exist also in the long run. It illustrates that the first adopters are the largest and most productive manufacturing firms. Further, since firms may have different risk assessments related to switching to software, there is a mix of software adopters and consultancy users in the middle range of firm size and productivity levels.

## 6 Conclusion

Economic history documents that the adoption of technology is gradual with long delays. Furthermore, it is amply documented in the business literature that the adoption of new technology in firms requires organisational changes and new skills, which constitute significant switching costs for individual firms. Nevertheless, recent literature on AI and the future of jobs overlooks or abstracts from such switching costs and assume that AI-based service automation technology is adopted as soon as it is invented, with dramatic effects on jobs. To understand, predict and prepare for the labour market implications of AI on jobs a much better grasp on what drives the *adoption* of technology is needed. Our paper contributes to filling this gap, studying the adoption of AI-based automation jointly in engineering services firms and their manufacturing customers.

Our simulations generate results that resonate with insights from economic history. First, AI-based automation, like general purpose technologies before, is adopted gradually. It starts at a slow pace, and accelerates after reaching a critical mass of adoption. Second, switching costs on the user side is the most important factor holding back the adoption of new technology. Third, technology does indeed destruct jobs, but it also generates new high-skilled jobs in the technology-using sectors. Finally, our simulations generate a boom and bust cycle on the supply side of the technology sector, which resembles what we have observed in the past, for instance during the *dot.com* bubble. This is not often observed in the literature and is thus an important contribution to new insight.

A policy implication of our findings is that innovation policy is not enough to foster technical progress. New technology is of little use if it is not adopted. We find that the early adopters are the largest and most productive manufacturing firms and that network effects of technology adoption can be strong. Furthermore, we find that adoption of AI-based automation is associated with demand for more skilled labour in using sectors. Policies aiming at fostering technical progress therefore need to focus more on switching costs on the user-side and on education and skills to make sure that the potential users of new technology can find the skills needed to restructure production around the technology.

The importance of the demand-side also suggest that occupational licensing does not necessarily constitute a drag on technology adoption as long as at least one engineering firm offers software. However, if exclusive rights to offer a service extends to software that automates the same service, the license fee is likely to be higher than in a competitive market, and the adoption rate may be substantially slowed down.

Finally, our results are relevant for other occupations and sectors. First, AI-enabled automation software in engineering is also relevant for the construction sector in a similar manner as in manufacturing. Second, other high-skilled business services occupations such as architects and management consultants face similar technological changes as the ones simulated here for engineering. Although these professions are currently way behind engineering in using AI-based automation, they are susceptible to such automation in the future. The accelerated digital transformation during the Covid-19 crisis may, however, have brought us closer to the steep part of the adoption curve for some of these services. Developments in the engineering sector modelled in this paper could thus be a harbinger of things to come in other professions going forward.

## Data Availability

The raw data supporting the conclusion of this article will be made available by the authors, without undue reservation.

## Footnotes

1 Over the past 200 years in 166 countries it has taken 45 years on average from innovation to adoption of technology (Comin and Hobijn, 2010).

2 See https://www.scb.se/contentassets/4d9059ef459e407ba1aa71683fcbd807/uf0301_2019a01_br_xftbr2001.pdf for Sweden and Zolas et al. (2021) for the US.

3 Accessibility of AI technology is not just improved by platforms for Machine Learning or Deep Learning, such as (Keras or Pytorch), but also by initiatives such as OpenAI https://beta.openai.com/or AI4EU https://www.ai4eu.eu/.

4 See for instance (Baldwin and Forslid, 2020).

5 see https://ec.europa.eu/eurostat/web/main/data/database.

6 See https://digital-strategy.ec.europa.eu/en/library/european-enterprise-survey-use-technologies-based-artificial-intelligence.

7 See also (Geroski, 2000) for a review of the literature.

8 Such complementary investment can be up to an order of magnitude larger than the initial investment in technology such as computers and other information technology (Milgrom and Roberts, 1990; Brynjolfsson and Hitt, 2000; Bessen, 2002; Bresnahan et al., 2002).

9 There is ample evidence that ICT and AI complement skills in the workplace. See for instance (Berman et al., 1998; Autor et al., 2003; Bessen et al., 2018; Brynjolfsson et al., 2019).

10 This is close to empirical estimates of the shape parameter of productivity distribution from firm level data (Feenstra, 2018). A shape parameter larger than two ensures that the variance of the distribution can be identified.

11 The figure is drawn for the parameter values depicted in Table 2.

12 International trade in engineering services would also limit the ability to charge a high mark-up and thus spur innovation. Adding space and different wages across countries could further exploit this point in future work.

13 By explicitly integrating the software lifecycle into the model, we also capture the idea that technology is not static, but needs to be updated from time to time.

14 We labelled the phases after analysing all simulation runs, also illustrating the shared observations. All experiments start with a situation in which all engineers are employed at engineering companies who exclusively offer consultancy services.

15 The parameter values reflect empirical relations observed in OECD countries. Wages of production workers are the numeraire in the model and set to unity. Data on employment by occupation and sector is not readily available, but the share of university educated workers in the total labour force is about 20% in the EU and 23% in the US https://ilostat.ilo.org/topics/employment/. The wage premium for professionals relative to plant and machine operators was about 1.4 in Sweden and 1.7 in the US in 2019 according to ILO statistics. https://ilostat.ilo.org/topics/wages/. Technicians and associate professionals account for 16% of all employees in manufacturing in the European Union, while computer, mathematical, architecture and engineering professionals account for another 9% https://ec.europa.eu/eurostat/databrowser/view/lfsa_eisn2/default/table?lang=en. The mark-up rate is also consistent with rates in the literature, while the other parameters are used to calibrate the model and to experiment with different scenarios.

16
www.simsesam.org.

17 We repeat every simulation 30 times. If not otherwise stated, diagrams show averaged values. Where suitable, we also give the standard distribution which is naturally higher in the transient phase 2 and low in phase 3.

18 Technically, this may also be modelled as an exit of the firms that fail to sell the software they have developed, while start-up engineering firms use the consultancy model, or as a single firm switching between business models. The results are the same either way.

19 Recall that the model captures innovation using existing AI technology to develop software. Considering software evolution with explicit software maintenance does not account for technological game change, rather for small, yet continuous improvements. Had the underlying AI-technology changed, a steady state might not occur. The authors thank an anonymous referee for making this point.

20 See for instance (Doms et al., 2004) for a study of the *dot.com* bubble in the United States. Our model does not have a financial market, but still generates a boom-bust cycle due to expectations and herd behaviour. The authors thank an anonymous referee for making this point.

21 Our model has a fixed number of workers and engineers. As software and learning by doing reduces the unit cost of production, employment in manufacturing and engineering firms may decline and unemployed workers may seek work in other sectors. Transition of workers to other sectors is not directly captured by our model. However, by keeping wages fixed, we implicitly capture an outside option at the going wage for workers.

22 Unfortunately, more detailed employment data by occupation and sector is not publicly available. However, EU aggregates should be representative for developed countries.

23 There are parameter values where this may not be the case. These are however outside the scope of what is reasonable considering available data.

## References

[B1] Acemoglu D. Restrepo P. (2018). The Race between Man and Machine: Implications of Technology for Growth, Factor Shares, and Employment. Am. Econ. Rev. 108, 1488–1542. 10.1257/aer.20160696

[B2] Andrews D. Criscuolo C. Gal P. N. (2015). Frontier Firms, Technology Diffusion and Public Policy: Micro Evidence from OECD Countries. OECD Productivity Working Papers, 2015-02. Paris:OECD Publishing. 10.1787/5jrql2q2jj7b-en

[B3] Autor D. H. Levy F. Murnane R. J. (2003). The Skill Content of Recent Technological Change: An Empirical Exploration. Q. J. Econ. 118, 1279–1333. 10.1162/003355303322552801

[B4] Baldwin R. Forslid R. (2020). Globotics and Development: When Manufacturing is Jobless and Services are Tradable. Tech. Rep. Cambridge, MA: National Bureau of Economic Research. 10.3386/w26731

[B5] Berman E. Bound J. Machin S. (1998). Implications of Skill-Biased Technological Change: International Evidence. Q. J. Econ. 113, 1245–1279. 10.1162/003355398555892

[B6] Bessen J. E. Impink S. M. Reichensperger L. Seamans R. (2018). The Business of Ai Startups. Boston: Boston Univ. School of Law, Law and Economics Research Paper. 10.3386/w24235

[B7] Bessen J. (2002). Technology adoption Costs and Productivity Growth: The Transition to information Technology. Rev. Econ. Dyn. 5, 443–469. 10.1006/redy.2001.0152

[B8] Bresnahan T. F. Brynjolfsson E. Hitt L. M. (2002). Information Technology, Workplace Organization, and the Demand for Skilled Labor: Firm-Level Evidence. Q. J. Econ. 117, 339–376. 10.1162/003355302753399526

[B9] Brynjolfsson E. Hitt L. M. (2000). Beyond Computation: Information Technology, Organizational Transformation and Business Performance. J. Econ. Perspect. 14, 23–48. 10.1257/jep.14.4.23

[B10] Brynjolfsson E. Rock D. Syverson C. (2019). “Artificial intelligence and the Modern Productivity Paradox“, in The Economics of Artificial Intelligence an Agenda. Editors A. Agrawal, J. Gans, and A. Goldfarb (Chicago: University of Chicago Press) 23–57.

[B11] Comin D. Hobijn B. (2010). An Exploration of Technology Diffusion. Am. Econ. Rev. 100, 2031–205910. 10.1257/aer.100.5.2031

[B12] Davies S. Davies G. (1979). The Diffusion of Process Innovations. (Cambridge, United Kingdom: CUP Archive).

[B13] Dawid H. (2006). Chapter 25 Agent-Based Models of Innovation and Technological Change. Handbook Comput. Econ. 2, 1235–1272. 10.1016/s1574-0021(05)02025-3

[B14] Gatti D. D. Fagiolo G. Gallegati M. Richiardi M. Russo A. (2018). Agent-Based Models in Economics - A Toolkit. (Cambridge, United States: Cambridge University Press).

[B15] Doms M. (2004). The Boom and the Bust in information Technology investment. San Francisco, United States: Economic Review-Federal Reserve Bank of San Francisco, 19–34.

[B16] Farmer J. D. Foley D. (2009). The Economy Needs agent-Based Modelling. Nature 460, 685–686. 10.1038/460685a 19661896

[B17] Feenstra R. C. (2018). Restoring the Product Variety and Pro-competitive Gains from Trade with Heterogeneous Firms and Bounded Productivity. J. Int. Econ. 110, 16–27. 10.1016/j.jinteco.2017.10.003

[B18] Felten E. W. Raj M. Seamans R. (2019). The Occupational Impact of Artificial Intelligence: Labor, Skills, and Polarization. New York, NY: NYU Stern School of Business.

[B19] Gallegati M. Richiardi M. G. (2009). “Agent Based Models in Economics and Complexity,” in Encyclopedia of Complexity and Systems Science. Editor Meyers R. A. (New York, NY: Springer New York), 200–224. 10.1007/978-0-387-30440-3_14

[B20] Geroski P. A. (2000). Models of Technology Diffusion. Res. Pol. 29, 603–625. 10.1016/s0048-7333(99)00092-x

[B21] Gort M. Klepper S. (1982). Time Paths in the Diffusion of Product innovations. Econ. J. 92, 630–653. 10.2307/2232554

[B22] Hall B. H. Khan B. (2003). Adoption of New Technology. Tech. Rep. Cambridge, United States: National bureau of economic research. 10.3386/w9730

[B23] Hamill L. Gilbert N. (2016). Agent-Based Modelling in Economics. New York, United States: John Wiley & Sons.

[B24] Klügl F. Bazzan A. L. C. (2012). Agent-based Modeling and Simulation. AIMag 33, 29. 10.1609/aimag.v33i3.2425

[B25] Lasi H. Fettke P. Kemper H.-G. Feld T. Hoffmann M. (2014). Industry 4.0. Bus Inf. Syst. Eng. 6, 239–242. 10.1007/s12599-014-0334-4

[B26] Milgrom P. Roberts J. (1990). The Economics of Modern Manufacturing: Technology, Strategy, and Organization. Am. Econ. Rev. 80, 511–528.

[B27] Nelson R. R. Phelps E. S. (1966). Investment in Humans, Technological Diffusion, and Economic Growth. Am. Econ. Rev. 56, 69–75.

[B28] Neves F. Campos P. Silva S. (2019). Innovation and Employment: An agent-Based approach. J. Artif. Societies Soc. Simulation 22. 8. 10.18564/jasss.3933

[B29] Rock D. (2019). Engineering Value: The Returns to Technological Talent and investments in artificial intelligence. Available at SSRN 3427412.

[B30] Rogers E. M. (1995). Diffusion of innovations: Modifications of a Model for Telecommunications. Die diffusion von innovationen in der telekommunikation. New York, United States: Springer, 25–38. 10.1007/978-3-642-79868-9_2

[B31] Rosenberg N. (1972). Factors affecting the Diffusion of Technology. Explorations Econ. Hist. 10, 3–33. 10.1016/0014-4983(72)90001-0

[B32] Stoneman P. L. David P. A. (1986). Adoption Subsidies vs information Provision as instruments of Technology Policy. Econ. J. 96, 142–150. 10.2307/2232977

[B33] Tesfatsion L. (2006). “Chapter 16 Agent-Based Computational Economics: A Constructive Approach to Economic Theory,”. Handbook of Computational Economics. Editors Tesfatsion L. Judd K. L.. 1 edn (Philadelphia, New York: Elsevier), 2, 831–880. 10.1016/s1574-0021(05)02016-2

[B34] Varian H. (2019). 16. Artificial Intelligence, Economics, and Industrial Organization. Chicago, United States: University of Chicago Press.

[B35] Wang J. Ma Y. Zhang L. Gao R. X. Wu D. (2018). Deep Learning for Smart Manufacturing: Methods and applications. J. Manufacturing Syst. 48, 144–156. 10.1016/j.jmsy.2018.01.003

[B36] Wooldridge M. (2009). An Introduction to Multiagent Systems. 2nd Edn. New York, United States: John Wiley & Sons.

[B37] Zolas N. Kroff Z. Brynjolfsson E. McElheran K. Beede D. N. Buffington C. (2021). Advanced Technologies Adoption and Use by US Firms: Evidence From the Annual Business Survey. Tech. Rep. New York, United States: National Bureau of Economic Research.

